# Fostering Students' Autonomy and Engagement in EFL Classroom Through Proximal Classroom Factors: Autonomy-Supportive Behaviors and Student-Teacher Relationships

**DOI:** 10.3389/fpsyg.2021.767079

**Published:** 2021-10-20

**Authors:** Kunni Han

**Affiliations:** ^1^College of Liberal Arts, Journalism and Communication, Qingdao University, Qingdao, China; ^2^Institute for Research on Portuguese-Speaking Countries, City University of Macau, Macau SAR, China

**Keywords:** EFL classroom, students' autonomy, students' engagement, teacher-student interaction, autonomy-supportive behaviors

## Abstract

Language learning achievement depends on student engagement which is at the center of attention these days. To assist students to become autonomous and independent learners, providing a social and supportive context is beneficial through autonomy-supportive and interaction. When learners are given the freedom to make choices about their education, they are likely to feel more enthusiastic and engaged. Moreover, learners' academic and social practices are largely influenced by educators, who play a major role as social agents and the function of the educators as the most dominant figures is the cornerstone of the language classroom. As there is a dearth of studies that have considered teachers and student interactions among all other effective issues and their significant effect on students' autonomy and engagement from the perspective of self-determination theory (autonomy support), the present review endeavors to focus on teacher-student interaction from the social perspective and their effects on student engagement in EFL classrooms. Subsequently, some implications are presented to elucidate the practice of teachers, students, teacher educators, materials developers.

## Introduction

Many teachers across the world, whether in language learning or other fields, have identified the challenges of keeping students engaged and focused on the solutions when they are faced with multiple distractions (Mercer and Dörnyei, 2020). Due to the potential of learners' engagement in resolving persistent instructive issues like a low accomplishment, high dropout rates, and high paces of learners' fatigue and aggression, there have been a bulk of investigations about engagement in the classroom in recent decades (Fredricks, 2015; Boekaerts, 2016). The degree of cooperation in instructing exercises is known as learners' engagement (Sun and Rueda, 2012). As engagement predicts students' drawn-out educational accomplishment, it fills in as a significant social indicator (Skinner et al., 2008). Investigating learners' engagement has expanded a range of hypothetical practices. To inspect the connections between context-oriented components, patterns of engagement, and change, a few researchers have utilized persuasive hypotheses like self-determination, self-guideline, flow, objective hypothesis, and expectance-value (Fredricks et al., 2016).

Self-determination theory (SDT) is an instructional theory of enthusiasm that is grounded on the essential value of student autonomy (Ryan and Deci, 2000). Along with the SDT, people have three important necessities: the necessity for autonomy, the necessity for capability, and the necessity for belongingness (Anja et al., 2016). As stated by Ryan and Deci (2017), how educators meet these fundamental necessities of learners will impact learners' prosperity, inspiration, engagement, and accomplishment. The experience of learners in the class during a semester can be useful and valuable if they see autonomy support from their educators, fulfill the mental necessity for autonomy, and involve during class discussion (Jang et al., 2016a).

SDT is regarded as a mediational paradigm in which the educator's instructing style in the class is fundamental and goes about as a mediator of class engagement. Thus, learners' fundamental mental necessities will be sustained and met when the educator gives autonomy support in the class, which, thus, will foresee the amount of class' engagement (Núñez and León, 2019). In their discussion of the SDT, Deci and Ryan (2016) argued that competence, autonomy support, and relatedness are among the most essential elements supporting learner autonomy. As they argue, when people engage themselves in a variety of activities socially, they feel relaxed and more connected to the community, and these processes lead to more control and autonomy throughout life. It is proposed that the connection between context-oriented attributes and learners' mental necessities impacts class engagement (Chen et al., 2021). The educator plays the role of a contextual facilitator of fulfilling learners' needs. Subsequently, the autonomy support that educators give upgrades learners' engagement as it helps fulfill learners' necessity for autonomy, which means experiencing a feeling of volition (Hospel and Galand, 2016).

Indeed, as declared by Skinner et al. (2008), autonomy has frequently been demonstrated as a huge indicator of changes in engagement. In case it is fulfilled, it prompts more constructive emotive, psychological, and behavioral results (Jang et al., 2012) and it is significantly linked to teacher success and accordingly learners' enthusiasm (Derakhshan et al., 2020).

Teacher-student interactions are among the remarkable aspects presumed to help student engagement in the classroom (e.g., Wang and Eccles, 2013; Furrer et al., 2014; Quin, 2017; Wang and Derakhshan, 2021; Xie and Derakhshan, 2021). The educational and social-emotional growth of learners is largely regulated by their classrooms (Hamre et al., 2013). Learners acquire knowledge and skills in their classes, learn social skills, and begin to develop a sense of self as they spend many hours there. As learners progress through the educational system, the events that occur in class may also directly affect their attitudes and social literacy (Skinner et al., 2009; Skinner and Pitzer, 2012). It is shown in the literature that social interaction is a fundamental factor affecting language learning (Hrastinski, 2008). While learners spend most of their time at school, everyday actions and interactions that occur in the classroom and outside have a noteworthy effect on students' success and engagement (Derakhshan et al., 2019; Derakhshan, 2021; Pishghadam et al., 2021).

The process of education is never isolated from its sociocultural context but is deeply interconnected. Therefore, learning is a function of both psychological abilities and social activities. Social and psychological aspects of classroom learning are interrelated. Taking part in activities of mutual interest with educators and other learners that build learning opportunities is the primary means through which learning occurs and engaged learners are more likely to study and share knowledge with others (Walqui, 2006). Social interactions can be classified into two categories, namely learner-learner and educator-learner interactions (Thoms and Eryilmaz, 2014; Vuopala et al., 2016). Educators have unlimited duties to motivate learners to be more dynamic and engaged in their learning (Chapman and Van Auken, 2001). As a consequence, establishing effective communication between educators and learners is essential (Liu and Wang, 2020).

The educator-learner relationship, which is commonly linked to the psychological needs of learners (Bakadorova and Raufelder, 2018; Froiland et al., 2019; Xie and Derakhshan, 2021), is one of the most significant factors in their development at school, which arises out of an active relationship between the educator and the particular learner (Sabol and Pianta, 2012). It has been observed that external elements like the educator-learner relationships can lead to a high level of engagement (Rimm-Kaufman et al., 2009). Some situations are more favorable to learner performance than others when it comes to educator-learner interaction (Ruzek et al., 2014). There are many types of educator-learner relationship quality, which can range from psychosocial support to cognitive and academic support (Pianta and Hamre, 2009). Language educators often guide their learners. With more practice on the task, the teacher step by step decreases the rate and level of support until the learner can complete it independently (Vygotsky, 1978). The gap between current developmental level and maximum potential for solving problems under educator's guidance with skilled colleagues is referred to as the Zone of Proximal Development (ZPD) (Lantolf and Appel, 1994 as cited in Danli, 2017). During supportive interaction within the ZPD, the learner improves the skills he or she requires to be successful. The amount of input and feedback reduces specificity as learners become more confident in the subject or skill, allowing them to acquire autonomous abilities in the process (Danli, 2017). To develop autonomy in learning and teaching, scaffolding, as a systematic educational method, illustrates how educators can influence and exert control over many aspects and phases of instructional processes (Benson, 2011). The goal of autonomous learning is to become independent within learners' individual ZPDs (Cross, 2003). Similarly, learners are anticipated to become autonomous objects containing groups of factors when they learn within the ZPD, so they look for learning methods tailored to suit their learning styles rather than sitting back and waiting for their educators' directions (Nosratinia and Zaker, 2014). As a result, scaffolding is fundamental to encouraging an autonomous learning process in EFL (Smith and Craig, 2013). The purpose of scaffolding is to develop a learning environment where language learners play the role of active seekers of knowledge rather than passive learners, allowing them to be fully involved in the learning process without extensive educator direction and control (Chen, 2020).

Earlier studies have proved a relationship between high-grade classroom relations and learner education, educational approaches, success, well-being, enthusiasm, and commitment (e.g., Allen et al., 2011, 2013; Roorda et al., 2011; Pianta et al., 2012). Grounded on the outcomes of these investigations, it is revealed that learners who consider educators as building mindful, well-organized learning conditions with exclusive requirements that are clear and reasonable are bound to report students' engagement. Moreover, high engagement is connected to higher participation and grades, which demonstrates an indirect connection between learners' impression of educator support and scholastic execution via students' engagement. For creating scholastic engagement and accomplishment, learners' connections with educators are important (Furrer et al., 2014). Therefore, the present theoretical review intends to show how teacher-student social interaction through the framework of SDT is related to learner autonomy and engagement.

## Review of Literature

### Learners' Engagement

Generally, in language education and educational studies and practices, one of the demands of engagement as a paradigm is that it can arrange for a comprehensive view of how learners ponder, perform, and feel in teaching contexts (Oga-Baldwin, 2019). Learners' engagement refers to the time learners are being effectively engaged with their classroom assignments and exercises (Lei et al., 2018) and it is also characterized as how much learners are occupied with learning in the conventional teaching cycle and alludes to the time, exertion, and energy they exert on instructive learning assignments (Chang et al., 2016). In addition, Hiver et al. (2021) theorized engagement as the step that a student is not only physically but also mentally engaged in accomplishing a language learning task.

Student engagement is regarded as a multidimensional concept that comprises behavioral, affective (emotional), intellectual (cognitive) engagement, and agentic engagement (Harbour et al., 2015; Chang et al., 2016; Lei et al., 2018). Behavioral engagement alludes to learners' activities and cooperation in their education, containing learning exercises, like their behavior, exertion, and association in-class learning exercises and schoolwork (Fredricks et al., 2004). Affective (Emotional) engagement alludes to learners' sentiments toward their institute, learning, and educators, as well as their mentalities toward teaching, sense of connectedness, identification with the school, and degrees of attentiveness, fatigue, and other feelings identified with school and learning (Hu et al., 2012). Moreover, intellectual, self-guideline, objective-coordinated, and learning techniques that learners use in scholarly assignments and learning measures are known as psychological (cognitive) engagement (Hart et al., 2011; Harbour et al., 2015; Quin, 2017; Lei et al., 2018). Finally, as asserted by Reeve (2013), the degree to which learners add to the progression of the education they get in terms of posing inquiries, communicating inclinations, and requesting what they need is known as argentic engagement. Every part plays its part in the inner elements of commitment (Skinner et al., 2008).

### Teacher' and Student' Social Interaction (Scaffolding and ZPD)

Educators are at the core of the teaching-learning development, and they play a crucial role in both activities who are responsible for leading students in the best direction through their profession (Friere, 1990, as cited in Hussain et al., 2013). In the classroom, however, educators have more than one role, which means not only supporting learners to be successful but also creating a positive environment and encouraging learners' interest and motivation for learning. Therefore, the teacher must be personally and professionally acquainted with the students, since these experiences make a significant contribution to the relationship between the educator and learner (Khan, 2011). Consequently, the role of a teacher can be fundamental to the effective teaching and learning of a foreign language (Da Luz, 2015). According to Camp (2011), the effectiveness of an educator can have a significant effect on a student's ability to learn. When a teacher-student relationship is strong, it creates students' psychological connections, which allows them to feel calm and confident in front of their classmates and educators. Good communication between an educator and his or her learners can be one of the elements which influence positive relationships. The ability to maintain understanding is made easier by effective communication (Pratolo, 2019).

Scaffolding is an instructional method that facilitates the student's participation in an educational activity by structuring a learning assignment, using conversation for direction, and providing hints to assist the learner (Celce-Murcia, 2001). Scaffolding has historically been closely associated with the concept of ZPD from a sociocultural point of view (Hammond and Gibbons, 2005). The ZPD is an essential part of the scaffolding construct as the basis for its interpretation (Verenikina, 2003). A learner's proficiency level is measured by how effectively they resolve problems under adult supervision or in cooperation with more proficient colleagues (Vygotsky, 1978).

Students need scaffolding to be successful during class discussions (Raes et al., 2012). A large number of learners, particularly beginners with little previous knowledge and expertise in a particular field, require specific guidance to make sense of content, good decisions, monitor their progress and adapt to new issues. As part of scaffolding, learners are encouraged to classify related objectives, follow and analyze growth toward those objectives, clarify discrepancies between current knowledge and concepts still to be discovered, and create and update artifacts (Hannafin et al., 2009). A variety of scaffolding methods may be employed during SCL, including asking discovery questions, receiving peer feedback, finding appropriate solutions, and providing specific instructions (Sharma and Hannafin, 2007; Weigend, 2014). In addition to peers and educators, scaffolding sources can include technology. If scaffolding sources are combined; the effects can be greater than if they are applied individually. The recent study by Roschelle et al. (2010) compared a mixed (peers and technology) scaffold with societal reasons to encourage peers to ask questions, explain their opinions, and give responses of their own.

### Autonomy Support

Autonomy is defined as being capable of making decisions based on one's perceptions of the world. Learners have the power of choice over their actions when they are autonomous since they can attribute their actions to an inner source of authority (Reeve et al., 2008). To be competent, learners must be influential in their constant communications with the societal milieu and be able to practice and apply their abilities in their daily lives. External factors give information about a person's competence or capabilities, as well as support for competence to encourage them (Ryan and Deci, 2000). In *situations* where learners feel secure as members of a community, they are more likely to be involved in relatedness and autonomous learning will grow (Deci and Ryan, 2000).

The autonomy support described by Reeve (2016) relates to the effort of providing instruction in a classroom environment that supports learners' requirements for autonomy and the relationship between educator and learner. To clarify, educators' behavior and attitudes are essential aspects that can be used in discovering, developing, and improving learners' natural motivational abilities. As suggested by Reeve (2016), the primary objective of autonomy support is to confirm and clarify that the learning process, classroom atmosphere, and the connection between educator and learner in ways that enhance autonomy. Listening to learners' ideas and providing a variety of educational opportunities, building their motivational skills, accepting their opinions, explaining how activities can be done, and talking in a non-obtrusive way with them are all behaviors that encourage freedom of choice. Having autonomy supported by the teacher results in improved motivation, interest in the classroom, learning motivation, and academic success (Ryan and Deci, 2017). By satisfying their needs, their engagement and motivation in classrooms are enhanced. The outcome is that they are more likely to enjoy better emotional and physical health, as well as perform more academically (Jang et al., 2016a,b).

## Implications and Future Directions

School leaders, teachers, and teacher educators can benefit from this review in a variety of ways. As a first step, the importance placed on the essential link between educator-learner interactions and learner motivation indicates that schools, educators, and teacher educators should evaluate how teacher-student interactions can be improved to care about learner engagement in the classroom. Second, there appears to be a link between educator-learner interactions and learner motivation. For positive engagement to be achieved, both of these factors should be applied, and they should be related to factors such as managing classes, ensuring the safety of learners, educational methods, and educator quality.

Through interaction between teacher and learners, opportunities were provided for them to encounter difficult tasks, to care for and inspire each other, to build an interactive context, and to have a positive outlook toward EFL autonomous learning. Indeed, students' autonomy can be deemed as the creation of interactive development and thus can be expanded through discussion (Little, 2007 as cited in Caixia, 2013). The ability of learners to be autonomous can be achieved through interactivity, which is characterized by clear instructions, adequate feedback, and direction, allowing learners to develop self-regulation and their ZPD ultimately. Having effective interaction means that both educators and learners appreciate how they can conduct the conversation. So, autonomy will improve (Danli, 2017).

Educators may assist learners' need for emotive connection and nurture their engagement through looking for prospects to cooperate with each learner, displaying particular attention, and providing well-being, encouragement, and help them in a suitable way (Pianta et al., 2012). Significantly, such constructive interaction and actions may also assist educators to get more satisfying emotional practices with their learners, leading to a more encouraging and optimistic classroom milieu and also it helps the growth of intervention databases to guide educators to care about students' autonomy.

Teacher trainers should be aware of how to be more autonomy-supportive in their classes and they should promote autonomy support by permitting learners to ask questions and attend in the discussion, debating multiple problem-solving tactics that all can be done through interaction that is a type of scaffolding and not only nurture autonomy in EFL context, but also support students' engagement and consequently achievement (Al-Issa, 2014). Moreover, through tasks that were used in the classroom through interaction, a positive atmosphere in the classroom may be built and constructed in a way that upsurge learners' engagement (Yu et al., 2019; Wang et al., 2021). This review of the literature shows that learners are more involved over time when educators keep close relationships with individual learners in the proximal classroom setting. The transition from one level to another seems to be especially challenging for learners. In addition, having strong emotional relationships with the educator may assist them in adjusting to changes in peer relationships, increasing responsibilities, and emotional demands, which may lead to the learners' engagement and achievement in class.

Educators can upgrade learners' engagement by giving ideal degrees of format and support for learners' autonomy, grounded on SDT (Hospel and Galand, 2016). Learners, who are locked in focus and partake in-class conversations, apply exertion in-class exercises and show awareness and inspiration to study (Fredricks et al., 2004). Also, they share thoughts, pose inquiries, and monitor each other's clues. In classes whose learners are involved, educators can unmistakably recognize what their learners comprehend and which ideas and points require more clarification and more profound conversation. Engaged learners who work in teams continue to examine, ask questions from one another and their educators, listen to one another with a critical ear, and contend with examples from their own lives and past information.

A learner who regularly credits a positive relationship with his/her educator in a class understands the material more quickly and acts well in the class. Logically, the nature of the relationship between the educator and the learner can be shaped and changed by both of their attributes. Hence, the more the learner is motivated by the educator, the better the learner will learn. Fostering a progressive association with learners is basic in succeeding the instructing learning cycle in a class since the positive connection between the two will encourage learners' participation and inspiration, and increment the learners' positive results at school (Varga, 2017). What increments openings for learners in acquiring the objectives of learning are the positive connections in the learning context. By providing choices and appropriate feedback on learners' autonomy, educators let learners take charge of their education.

In the courses taught by autonomy-supportive teachers through interaction, learners acquire and keep more knowledge for a longer period and demonstrate greater perseverance during learning (Reeve and Jang, 2006). Scaffolding is designed to enhance classroom learning which happens through educators providing appropriate feedback at the right time (Zhou and Lam, 2019). Consequently, educators who act as mediators to assist learners with overconfidence and excessively explicit instruction may hinder their progress in self-regulation. Thus, to monitor students' performance in the learning process, educators need to offer appropriate and adequate support. Mediation is a process that needs to be appropriated by learners to improve their capability to control their behavior and to be autonomous learners.

The study illustrates the potential value of educators facilitating scaffolding and supports the framework for considering the interdependence of autonomy and scaffolding. By the use of scaffolding s in communicative activities in the classroom context, educators can play the role of managers or motivators (Chen, 2020). In the interaction between educator and learner, it was discussed how the educator's support can facilitate efficient language practice and give learners the chance to discuss meaning and form in communication. In the scaffolding process (Vygotsky, 1978), first classroom goals were examined and then progressed to the development of learners within the ZPD model, which has shown the power of autonomy. Future studies could follow the concepts presented in this review in a form of empirical structure to better realize the probable relations between the students' and teachers' interactions and other classroom factors.

## Author Contributions

The author confirms being the sole contributor of this work and has approved it for publication.

## Conflict of Interest

The author declares that the research was conducted in the absence of any commercial or financial relationships that could be construed as a potential conflict of interest.

## Publisher's Note

All claims expressed in this article are solely those of the authors and do not necessarily represent those of their affiliated organizations, or those of the publisher, the editors and the reviewers. Any product that may be evaluated in this article, or claim that may be made by its manufacturer, is not guaranteed or endorsed by the publisher.
